# CONSUMPTION OF ULTRA-PROCESSED FOODS BY CHILDREN UNDER 24 MONTHS OF AGE AND ASSOCIATED FACTORS

**DOI:** 10.1590/1984-0462/2020/38/2018277

**Published:** 2020-02-14

**Authors:** Wanessa Casteluber Lopes, Lucinéia de Pinho, Antônio Prates Caldeira, Angelina do Carmo Lessa

**Affiliations:** a Universidade Federal dos Vales do Jequitinhonha e Mucuri, Diamantina, MG, Brazil.; b Universidade Estadual de Montes Claros, Montes Claros, MG, Brazil.

**Keywords:** Complementary feeding, Infant nutrition, Industrialized foods, Alimentação complementar, Nutrição infantil, Alimentos industrializados

## Abstract

**Objective:**

To evaluate the intake of ultra-processed foods by children under 24 months of age from the city of Montes Claros and identify factors associated with this consumption.

**Methods:**

This is a population-based cross-sectional study with data collected from households through interviews. A questionnaire assessed the sociodemographic conditions of the family, maternal and child characteristics, and food consumption. We adopted a multivariate model to identify factors associated with the intake of ultra-processed foods.

**Results:**

A total of 545 children participated in this study, of whom 74.3% consumed some kind of ultra-processed food. The factors most strongly associated with this consumption were children older than six months, infants who were not breastfed, households with up to three residents, and the main caregiver of the child being someone other than the mother.

**Conclusions:**

Children under 24 months start consuming ultra-processed products at an early age, replacing foods considered natural and healthy. This study can contribute to guide health professionals in counseling families about feeding in the first years of life, emphasizing the proper introduction of complementary feeding and discouraging the consumption of ultra-processed products.

## RESUMO

**Objetivo:**

Avaliar o consumo de alimentos ultraprocessados por crianças do município de Montes Claros, com idade inferior a 24 meses de idade, e identificar fatores associados a esse consumo.

**Métodos:**

Estudo transversal de base populacional, no qual a coleta de dados foi realizada nos domicílios, por meio de entrevistas. Aplicou-se um questionário para avaliar a situação sociodemográfica da família, as características materno-infantis e o consumo alimentar. Foi utilizado modelo multivariado para identificar fatores associados ao consumo de alimentos ultraprocessados.

**Resultados:**

Participaram do estudo 545 crianças, das quais 74,3% consumiam algum alimento ultraprocessado. Os fatores mais fortemente associados a esse consumo foram crianças com idade superior a seis meses de idade, que não faziam uso de leite materno, domicílios com até três habitantes e o principal cuidador da criança apontado como outros sem ser a mãe.

**Conclusões:**

O consumo de alimentos ultraprocessados está presente precocemente na alimentação das crianças menores de 24 meses de idade, substituindo alimentos considerados naturais e saudáveis. Este estudo poderá contribuir para a orientação dos profissionais de saúde no aconselhamento das famílias para a alimentação nos primeiros anos de vida, com ênfase na introdução adequada da alimentação complementar e desestimulando a introdução de produtos ultraprocessados.

## INTRODUCTION

In recent years, dietary patterns have changed in most countries, mainly due to the substitution of fresh or minimally processed foods for processed and ultra-processed products.^1^Among the conditions that favored this trend is the greater participation of women in the labor market, with consequent reduction in the time available to prepare meals; globalization; the socioeconomic development, which expanded the access to ready-made foods; the influence of the media in changing the eating habits of the population^1^ ^,^ ^2^ and the persuasive strategies used by the food industry through marketing; and the excessive increase in palatability, which can lead to addiction and uncontrolled appetite.^3^

The increasing intake of ultra-processed foods is one of the leading causes of the current pandemic of obesity and non-communicable diseases,^4^ ^,^ ^5^ as these products are more caloric, have larger amounts of free sugar, sodium, and total and saturated fats, and lower protein and fiber content, when compared to fresh or minimally processed foods.^5^ ^,^ ^6^ This modification in eating habits has contributed to change the Brazilian nutritional epidemiological profile, a process known as nutritional transition, in which diseases related to overweight, such as obesity, diabetes, and hypertension, overcame those associated with nutritional deficiencies.^7^

When introduced during childhood, ultra-processed foods, which have high energetic and low-nutritional values, reduce the immunological protection and can trigger allergic processes, hindering the digestion and absorption of nutrients, as well as the growth and development of the child.^2^ In Brazil, the Ministry of Health, following the guidelines from the World Health Organization (WHO), has elaborated the Dietary Guidelines for Children under Two Years, with recommendations called “Ten steps to a healthy diet for children under two years,” in which the eighth step advocates that sugar, coffee, canned foods, fried foods, soft drinks, hard candies, snacks, and other foods with large amounts of sugar, fat, and coloring agents should be avoided in the first years of life.^8^

Few studies have addressed food consumption taking into account the changes resulting from food processing, despite these changes affecting the health of individuals.^6^ ^,^ ^9^ Considering the increasing participation of processed foods in the diet of the population and the risks associated with their consumption, both in the short and long term, assessing the intake of ultra-processed products becomes necessary, particularly among infants, who constitute a vulnerable group.^2^ ^,^ ^4^ ^,^ ^6^ ^,^ ^9^ Thus, this study aimed to evaluate the intake of ultra-processed foods by children under 24 months of age from the city of Montes Claros and identify factors associated with this consumption.

## METHOD

This is a population-based analytical cross-sectional study, based on data from the study “Infant feeding in the first two years of life,”^10^ conducted in 2015. The target population consisted of children under 24 months living in the urban area of the city under study.

A probabilistic sample of permanent private households (PPHs) in the urban area of Montes Claros was selected in two stages (census tracts and blocks). The sample size was determined based on a 50% estimate of prevalence of the studied event (early weaning), adopting a 5% error and a correction factor of 1.5 for the design effect (deff). The sample population had a 10% increase to compensate for possible losses, resulting in a minimum initial sample size of 427 children. In the first stage, among the 385 census tracts listed in the 2010 Geographical Operational Base (*Base Operacional Geográfica* - BOG) of the Brazilian Institute of Geography and Statistics (*Instituto Brasileiro de Geografia e Estatística* - IBGE), 64 were systematically selected, with probability proportional to the number of PPHs in the 2010 Demographic Census. In the second stage, the blocks visited in each census tract were randomly chosen, including all children under 24 months living in the households. When the household selected had no children in the age group under study, new homes were chosen, following the order of a prior drawing. The visits gathered information from 545 children. The statistical power for the number of elements in the sample was estimated between 80 and 90%.

Data was collected by interviewing the caregivers of the children. The instrument used for this collection was a structured questionnaire, which covered the sociodemographic conditions of the family, maternal characteristics, child assistance and care, the child’s characteristics, and infant food consumption. A 24-hour dietary recall was administered to identify food intake. This instrument investigates the foods and beverages consumed by the child on the day prior to the interview and the food frequency, listing some ultra-processed foods. The “new classification” proposed by Monteiro et al.^11^ was adopted to classify ultra-processed foods; this methodology comprises the following groups: fresh or minimally processed foods, processed culinary ingredients, and processed and ultra-processed foods. The production of ultra-processed foods involves several steps, processing techniques, and ingredients, including salt, sugar, oils and fats, and substances for industrial use only, such as soy and milk proteins, meat extracts, elements obtained from the additional processing of oils, fats, carbohydrates, and proteins, as well as substances synthesized in laboratory from foods and other organic sources, like petroleum and coal.^12^ The present study considered ultra-processed foods the following products: soft drinks and processed juices, instant noodles, cookies, crackers, packaged snacks, candies (hard candy, toffees, lollipops), chocolate milk in general, *petit suisse* cheese, sweetened and flavored yogurts, and breakfast cereals. Reference to any one of these foods in the 24-hour dietary recall or the food frequency investigation was deemed a positive response for the intake of ultra-processed foods. This study did not include infant formula in the ultra-processed food group because this product is recommended for infants as a substitute for breast milk when it cannot be consumed.

**Figure 1:**
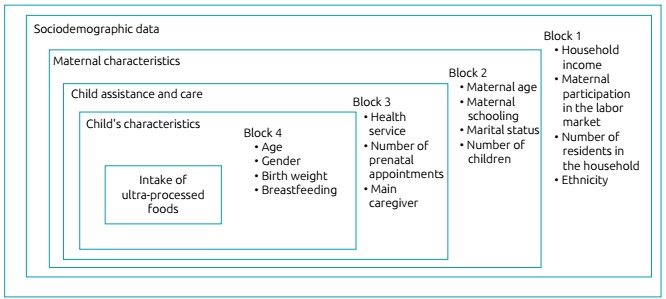
Hierarchical model proposed to assess the association between the intake of ultra-processed foods and characteristics of the family and the children aged 0-24 months.

We elaborated a theoretical hierarchical conceptual model (Figure 1) to identify the factors associated with the consumption of ultra-processed foods, based on the available literature.^13^ In this theoretical model, the distal hierarchical level consisted of sociodemographic variables, as these characteristics can influence the variables present in the following hierarchical levels, such as child care and characteristics of the mother and child. The dependent variable was the intake of ultra-processed foods by children, categorized as yes or no. As to the independent variables, obtained from the structured questionnaire with specific questions, we considered the following groups distributed in hierarchical levels:

- Sociodemographic data: household income, maternal participation in the labor market, and the number of residents in the household.
- Maternal characteristics: age, schooling, marital status, number of children, and ethnicity.
- Child assistance and care: type of health service used, number of prenatal appointments, and main caregiver.
- Child’s characteristics: age, gender, birth weight, and if the infant is breastfed.

Initially, we performed a descriptive analysis of the characteristics of the children and their families using absolute and relative frequency distributions. Next, we used Poisson regression with a robust variance to estimate crude prevalence ratios (PR) between independent variables and the outcome. At this stage, we selected those that presented a minimum significance level of 20% (p<0.20) for the multivariate model.

In the final model adjusted for each level, the only variables that remained were the ones with p<0.05 after control by variables of the same block and those that proved to be significant in hierarchically superior blocks. Statistical analyses were performed with the software Statistical Package for Social Sciences (SPSS), version 21.0, adopting a 95% confidence interval (95%CI).

The Research Ethics Committee of the Universidade Estadual de Montes Claros (REC/Unimontes) considered and approved this project, according to the Report no. 798,122, and all guardians signed the informed consent form.

## RESULTS

A total of 545 children participated in this study. Most infants were males, with appropriate birth weight, were breastfed, and older than 12 months of age. Regarding child care, most participants were followed in the public health service, with their mother as the only caregiver (Table 1).

**Table 1:** Demographic, socioeconomic, and behavioral characteristics of the family and the children aged 0-24 months.

Variables	N	%
Household income		
< 2 times the minimum wage	149	32.7
> 2 times the minimum wage	306	67.3
Maternal participation in the labor market		
Does not work outside the home	363	67.0
Works outside the home	179	33.0
Number of residents in the household		
≤ 3 residents	185	34.3
4-5 residents	258	47.9
≥ 6 residents	96	17.8
Maternal ethnicity		
White	225	41.6
Black	40	7.4
Multiracial/Asian	276	51.0
Maternal age		
< 20 years	83	15.4
20-34 years	383	70.9
> 35 years	74	13.7
Maternal schooling		
< 12 years of study	454	85.2
> 12 years of study	79	14.8
Marital status		
Single/widow	125	22.9
Married/domestic partnership	420	77.1
Number of children		
1 child	278	51.0
> 1 child	267	49.0
Health service		
Public	402	73.9
Private	142	26.1
Number of prenatal appointments		
1-5 appointments	47	10.1
≥ 6 appointments	420	89.9
Main caregiver		
Only the mother	367	67.4
Mother+others	89	16.3
Others	89	16.3
Child’s age		
< 6 months	130	23.9
> 6 to < 12 months	162	29.7
> 12 months	253	46.4
Child’s gender		
Female	251	46.1
Male	293	53.9
Birth weight		
< 2,500 g	45	8.4
> 2,500 g	493	91.6
Breastfeeding		
Yes	322	59.6
No	218	40.4

With respect to maternal and sociodemographic characteristics, over half of the mothers did not work outside the home, had a household income equal to or greater than 2 times the minimum wage, belonged to the age group 20 to 34 years, self-reported being multiracial, was married and/or in a domestic partnership, had less than 12 years of study, declared that 4 to 5 people lived in the household, and had only one child (Table 1).

**Chart 1:**
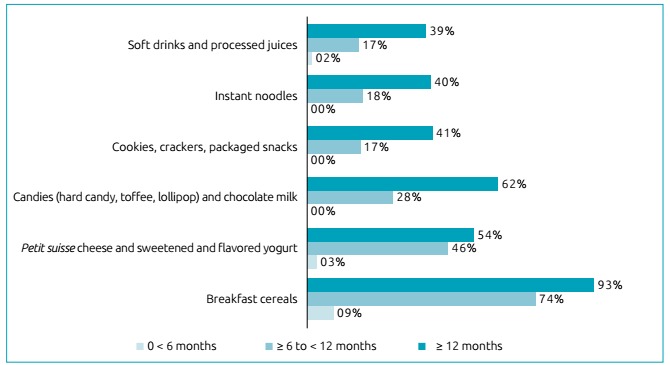
Main ultra-processed foods consumed by children aged 0-24 months in the city of Montes Claros, Minas Gerais.

Food intake analysis showed that 74.3% (n=405) of the children consumed some kind of ultra-processed food, most infants older than six months already made use of breakfast cereals, and half of them ate *petit suisse* cheese and sweetened and flavored yogurt, as shown in Chart 1.

As to the factors associated with the intake of ultra-processed foods, the following variables remained statistically significant in the multivariate analysis after adjustment according to the hierarchical model: households with up to 3 residents (PR 1.17; 95%CI 1.00-1.38), main caregiver of the child being someone other than the mother (PR 1.20; 95%CI 1.08-1.34), children over 6 months of age (PR 7.08; 95%CI 4.36-11.49) and 12 months of age (PR 7.83; 95%CI 4.80-12.76), and infants who were not breastfed (PR 1.12; 95%CI 1.04-1.20) (Table 2).

**Table 2:** Prevalence ratio of non-adjusted and adjusted analyses between characteristics of children aged 0-24 months and the consumption of ultra-processed foods per hierarchical levels.

Variables	Non-adjusted		Adjusted	
	PR (95%CI)	p-value	PR (95%CI)	p-value
Block 1				
Household income				
< 2 times the minimum wage	1	0.340		
> 2 times the minimum wage	0.94 (0.85-1.05)			
Maternal participation in the labor market				
Does not work outside the home	1	0.400		
Works outside the home	1.04 (0.94-1.15)			
Number of residents in the household				
≤ 3 residents	1.17 (1.01-1.38)	0.040	1.17 (1.01-1.38)	0.040
4-5 residents	1.11 (0.95-1.38)		1.11 (0.95-1.38)	
≥ 6 residents	1		1	
Maternal ethnicity				
White	1.05 (0.95-1.16)	0.330		
Black	1.00 (0.81-1.22)			
Multiracial/Asian	1			
Block 2				
Maternal age				
< 20 years	0.908 (0.74-1.10)	0.345		
20-34 years	1.019 (0.88-1.17)			
> 35 years	1			
Maternal schooling				
< 12 years of study	0.95 (0.84-1.09)	0.520		
> 12 years of study	1			
Marital status				
Single/widow	0.96 (0.84-1.08)	0.510		
Married/domestic partnership	1			
Number of children				
1 child	1.09 (0.99-1.21)	0.060		
> 1 child	1			
Block 3				
Health service				
Public	1	0.010		
Private	1.14 (1.03-1.25)			
Number of prenatal appointments				
1-5 appointments	1.05 (0.89-1.24)	0.530		
≥ 6 appointments	1			
Main caregiver				
Only the mother	1	<0.010	1	<0.010
Mother+others	1.11 (0.98-1.26)		1.08 (0.95-1.24)	
Others	1.21 (1.08-1.34)		1.20 (1.08-1.34)	
Block 4				
Child’s age				
< 6 months	1	<0.010	1	<0.010
> 6 to < 12 months	7.43 (4.60-12.01)		7.08 (4.36-11.49)	
> 12 months	8.59 (5.34-13.84)		7.83 (4.80-12.76)	
Child’s gender				
Female	1	0.410		
Male	0.96 (0.87-1.05)			
Birth weight				
< 2,500g	1.01 (0.85-1.20)	0.860		
> 2,500 g	1			
Breastfeeding				
Yes	1	<0.010	1	<0.010
No	1.60 (1.46-1.76)		1.12 (1.04-1.20)	

PR: prevalence ratio; 95%CI: 95% confidence interval.

## DISCUSSION

In this study, we identified a high intake of foods not recommended for the age group studied, demonstrating that children start consuming ultra-processed foods at a very early age. Other national studies show similar results. Freitas et al*.* ^14^ found the presence of soft drink (55.4%) and juice powder (63.9%) in the diet of children under one year of age in Porto Alegre, Rio Grande do Sul. Toloni et al.^2^ revealed similar results in a study conducted in the city of São Paulo, São Paulo, in which the introduction of soft drinks and processed juices to infants of the same age group of the present work occurred in 56.5 and 63.6% of cases, respectively. A study performed by Silveira, Neves, and Pinho,^15^ aiming at evaluating the diet of children enrolled in public daycare centers in the city of Montes Claros, Minas Gerais, also found high consumption of sweetened beverages, such as soft drinks and reconstituted juice powder. According to Lessa et al.,^16^ the ingestion of processed juices or juice powder is contraindicated for children in the first year of life, as they have food additives like tartrazine, which is associated with allergic reactions. The acceptable dietary intake (ADI) for this additive, defined by WHO experts, cannot be applied to children under 12 months of age because of their immature liver function.

Breakfast cereals, commonly used to thicken the milk, were present in the diet of most children over six months, while approximately 50% of infants in the same age group consumed *petit suisse* cheese/yogurt, which usually replaces a milk meal. Vitolo et al.^17^ conducted a study in Porto Alegre, Rio Grande do Sul, and found a prevalence above 70% of *petit suisse* cheese in the age group 6 to 15 months. These foods have a high concentration of sugar and are associated with the occurrence of dental caries and overweight.^17^ A study by Sparrenberger et al.^18^ showed a strong relationship between the intake of ultra-processed foods and obesity in children, as well as the presence of trans fat in these foods. The ingestion of these fats is associated with increased LDL-cholesterol levels, risk of cardiovascular disease, diabetes, and hypertension.^6^

The Ministry of Health recommends that sugar, coffee, canned foods, soft drinks, hard candies, snacks, and other candies should be avoided in the first years of life.^19^ The present study found that approximately 60% of the children over one year of age consumed candies and chocolate milk and 40%, cookies and packaged snacks. Other authors also identified a high consumption of ultra-processed products in the diet of infants.^20^ ^,^ ^21^

In the present study, we detected a higher intake of ultra-processed foods by children who lived in households with a smaller number of residents, whose main caregiver was not their mother, older than six months, and who were not breastfed. According to Campos et al.,^22^ the maternal role stands out in the context of public health policies due to their socially constructed characteristic of caregiver, having greater care with individual and family health. The same authors state that the importance of maternal care can also be identified in the family diet, mainly because most mothers are responsible for the household budget, shopping, and food preparation. A study on infant feeding conducted by Goes et al.^23^ in Lisbon, Portugal, reported greater involvement of mothers (93.1%) compared to fathers (6.9%). According to Silva, Costa, and Giugliani,^24^ the interaction with the person who feeds the child determines if the feeding will be responsive or not and will influence the child’s eating habits and relationship with food. Other studies carried out in Brazil report that help from a relative at home increases the risk of interrupting breastfeeding before the infant reaches four months of age.^25^ Campagnolo et al.^26^ revealed that child separation, due to the mother returning to work outside the home, is an independent risk factor for the early introduction of other beverages and foods.

The association found herein between the caregiver and the early introduction of ultra-processed foods needs to be considered from the perspective of programs aimed at promoting healthy eating habits for children. Child care, particularly regarding their feeding, is no longer solely attributed to the mother, probably due to the greater participation of women in the labor market, when compared with past decades. With respect to programs traditionally focused on mothers, they must rethink their strategy in order to reach the whole family.

The present study identified a higher consumption of ultra-processed foods by children in households with a lower number of residents. This finding could be an indirect measure of household income, despite this variable not remaining in the final model. Measurement of household income represents one of the main difficulties for epidemiological studies since families tend to provide wrong information about their income. Using indirect measures, such as the number of rooms, residents, or children at home, can be a valid strategy in these cases. Thus, the variable “number of residents in the household” could be a more accurate measure of household income in this study. In this case, we can reasonably assume that these families have a higher income available to purchase foods considered superfluous, such as packaged snacks, soft drinks, candies, among others. A study conducted by Levy et al.^27^ revealed that the consumption of “added sugars” derived from processed and ultra-processed foods increased with the elevation of household income.

This work identified a higher proportion of intake of ultra-processed foods among children over six months of age. A study developed by Batalha et al.^28^ presented a similar result when assessing the intake of ultra-processed foods by children aged 13 to 35 months. Sparrenberger et al.^18^ also found an association between the consumption of ultra-processed foods and increasing age. In this life stage, the infant begins to receive the food prepared for the family, and if ultra-processed foods are present in the diet of the family, the child will probably start to introduce these products to their diet as well. In this regard, Louzada et al.,^5^ when evaluating food acquisition in households of Brazilian metropolises between 1987 and 1988 and between 2008 and 2009, detected a systematic increase of ultra-processed foods in the diet of this population. Some authors have reported the influence of advertisement, especially the one broadcast by television, and daycare as vulnerable points for the early introduction of ultra-processed foods.^2^ ^,^ ^18^

The supply of foods considered unhealthy suggests an inadequate introduction of complementary feeding to infants, characterizing a risk factor for the reduction in the duration and frequency of breastfeeding, the acquisition of habits and predisposition for obesity, and the development of chronic non-communicable diseases in adulthood.^29^ The present study identified an association between weaning and the intake of ultra-processed foods. Caetano et al.^30^ evaluated the practices and food consumption of healthy infants in three Brazilian metropolises and found a short duration of breastfeeding and a high prevalence of processed foods in the child’s diet. Thus, among the widely known benefits of breastfeeding, the protection against the early introduction of ultra-processed foods should stand out.

The results of this study must be interpreted considering some limitations, including the data collected on food consumption being subject to memory bias, which could represent suppression of information about the foods consumed. Data generalization is also limited by the lack of information about some variables. Nonetheless, the results are important, as they derive from a representative sample of infants and for revealing a situation previously unknown in the region studied.

We can conclude that children under 24 months start consuming ultra-processed products at an early age, replacing foods considered natural and healthy. We identified the following factors associated with the early introduction of ultra-processed foods: households with up to three residents, children whose main caregiver is not their mother, those older than six months, and who are no longer breastfed. Therefore, this study can contribute to guide health professionals in counseling families about feeding in the first years of life, emphasizing the proper introduction of complementary feeding and discouraging the consumption of ultra-processed products.
